# Supplementary material for: Gelsolin and dCryAB act downstream of muscle identity genes and contribute to preventing muscle splitting and branching in Drosophila
Source: Sci Rep. 2021 Jun 23;11:13197. doi: 10.1038/s41598-021-92506-3 (PMC8222376; doi:10.1038/s41598-021-92506-3)

Supplemental information for the manuscript:

Gelsolin and *dCryAB* act downstream of muscle identity genes and contribute to preventing muscle splitting and branching in *Drosophila*

Benjamin Bertin, Yoan Renaud, Teresa Jagla, Guillaume Lavergne, Cristiana Dondi, Jean-Philippe Da Ponte, Guillaume Junion* and Krzysztof Jagla*

GReD Institute - INSERM U1103, CNRS UMR6293, Université Clermont Auvergne,
28, place Henri-Dunant, 63000 Clermont-Ferrand, France

Supplementary Figure legends and Supplementary figures:

Figure S1. Sensitivity assessment of translational profiling by TRAP.

A-B. Vulcano plots showing distribution of up- and down-regulated genes in Slou- and Lms-positive muscle subsets at the T3 time point. Note that genes known to be specifically expressed in the slou population (*slou, org-1*) and the lms population (lms) are consistently up-regulated. **C-D**. Distribution of Slou and Lms up-regulated and down-regulated genes from the T2 time window with respect to modEncode RNA expression level based on whole embryo RNA sequencing (stage 12–16).

Figure S2. *Gel* is expressed in LTs from stage 14 and is present in other mesodermal tissus. *Gel* expression in stage 13 (**A,A'**) and stage 14 (**B,B'**) embryos revealed by *in situ* hybridisation with the *Gel* RNA probe. Muscles are stained with anti-actin antibodies. *Gel* transcripts are first detected in LT muscles at stage 14 and are also found (**C,C'**) in visceral muscle (VM) and fat body (FB) (arrows).

Figure S3. Localisation of dCRyAB and Gel proteins in LT muscles. (A, A') In stage 15 embryos dCryAB displays a dotty cytoplasmic pattern in LTs and is detected close to the myotube membrane (arrows) and at LTs extremities (arrowheads). (**A'**) As revealed by muscle-specific Mef>LifeAct-GFP dCRyAB partially co-localizes with Actin in LTs. (**B,B'**) In stage 15 embryos Gel is highly enriched at the extremities of LT myotubes (arrowheads) but discrete Gel spots are also detected at sub-membrane LT areas (arrows). (**B'**) Colocalization of Gel and F-Actin is seen at the LT myotube ends.

Figure S4. Branched and split phenotypes in LT iTF mutant embryos. (A-C) Lateral views of four hemisegments of stage 16 embryos stained with anti-Actin (A, B) and anti-β3-Tubulin antibodies. (A) wild type muscle pattern. (B) An example of split (arrows) LT muscles in apterous *ap*^{UGO35} mutant embryos. Asterisks point to lacking LT muscles (C) An example of branched LT muscle phenotype (arrowheads) in *lms*^{S95} mutant embryo.

Figure S5. *In vivo* visualisation of LT splitting in *Gel* mutants.

Time lapse movie (3 h 30 min, with 3 min frame interval) from (A) control *lms>LAGFP and (B) Gel;lms>LAGFP* embryos showing one abdominal segment (A5) with developing LT muscles. The films begin at late stage 14 and end at late stage 16 and were generated using Imaris software version 9.2; URL: https://imaris.oxinst.com (B) Splitting of LT3 and LT4

muscles could be observed. It is initiated at stage 15 and fully apparent with split fibre morphology and duplicated ends at stage 16.

Figure S1

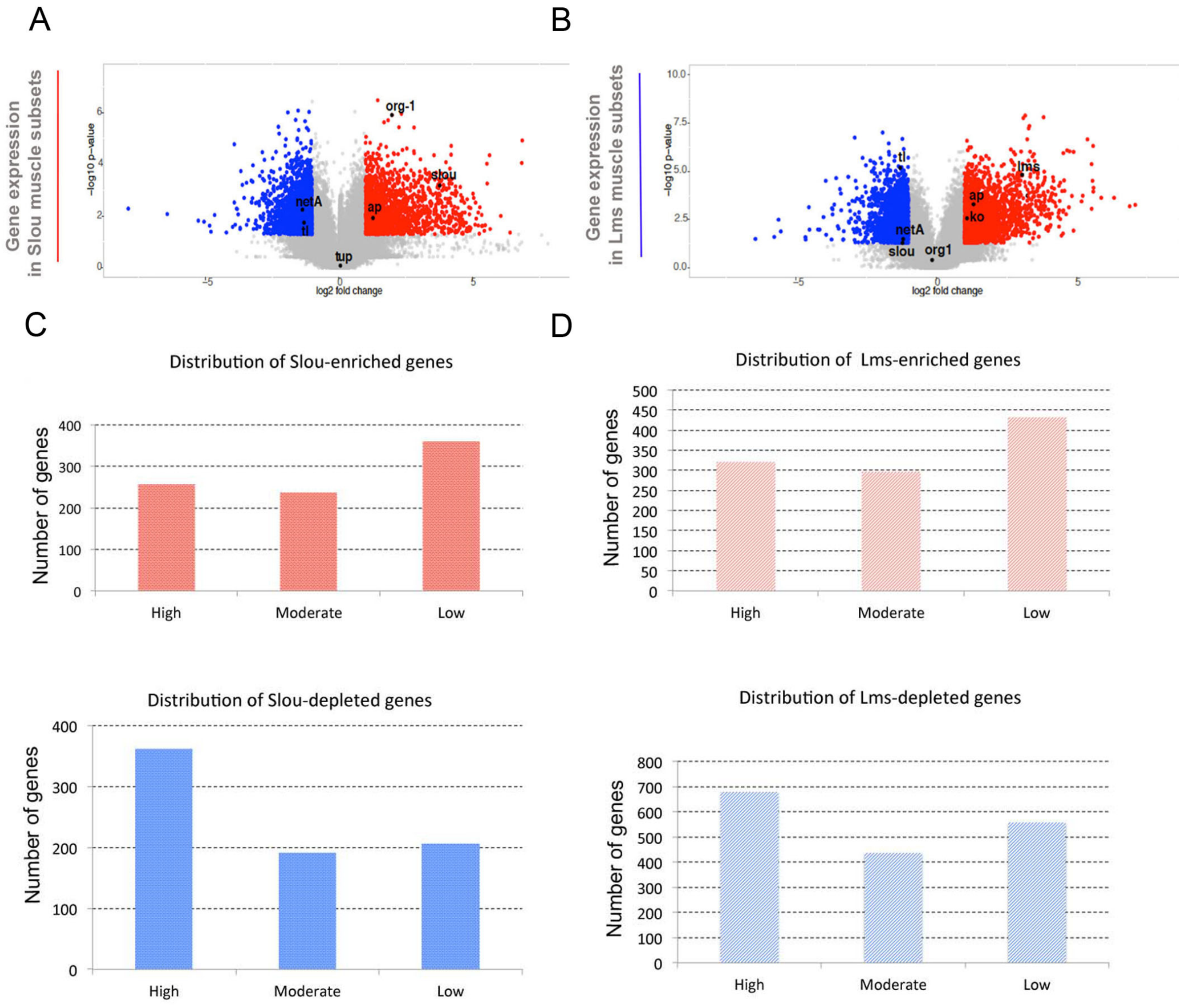

Figure S2.

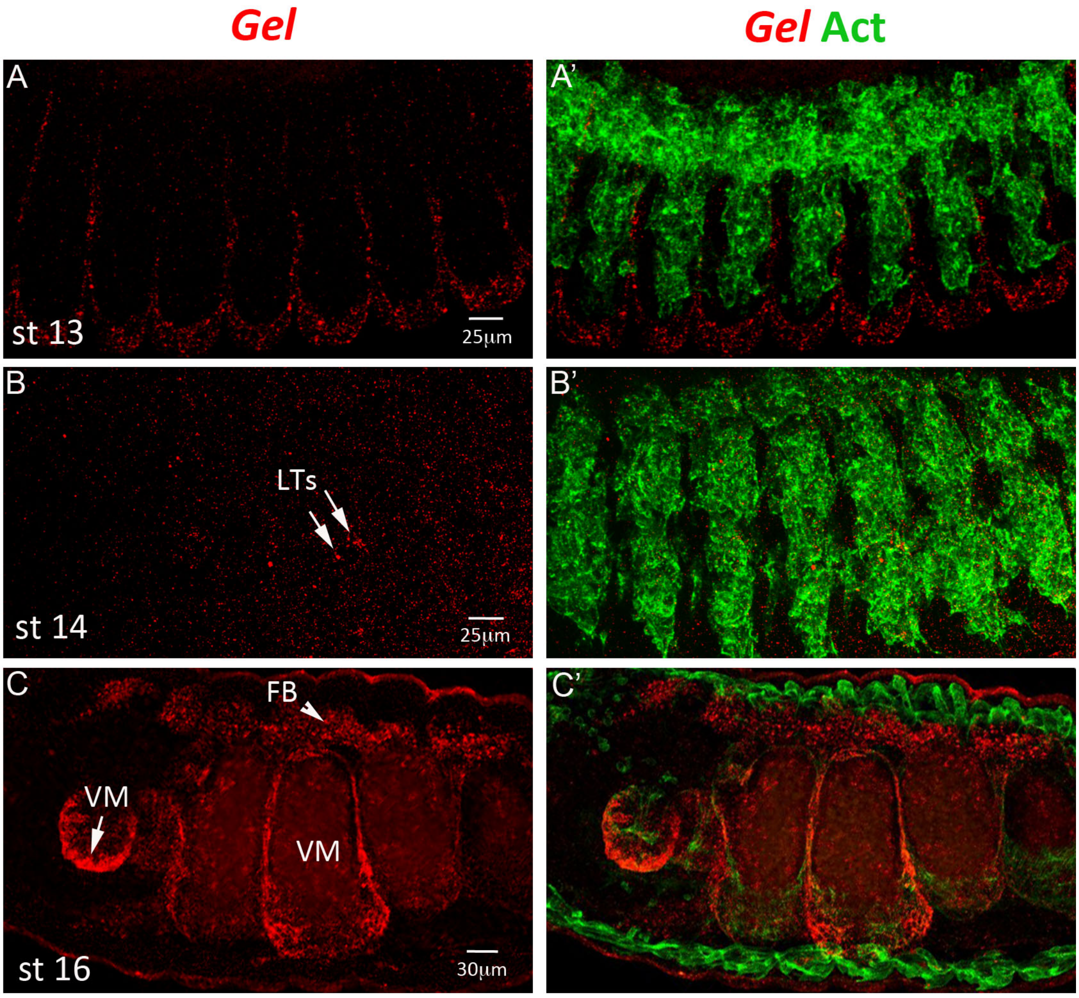

Figure S3

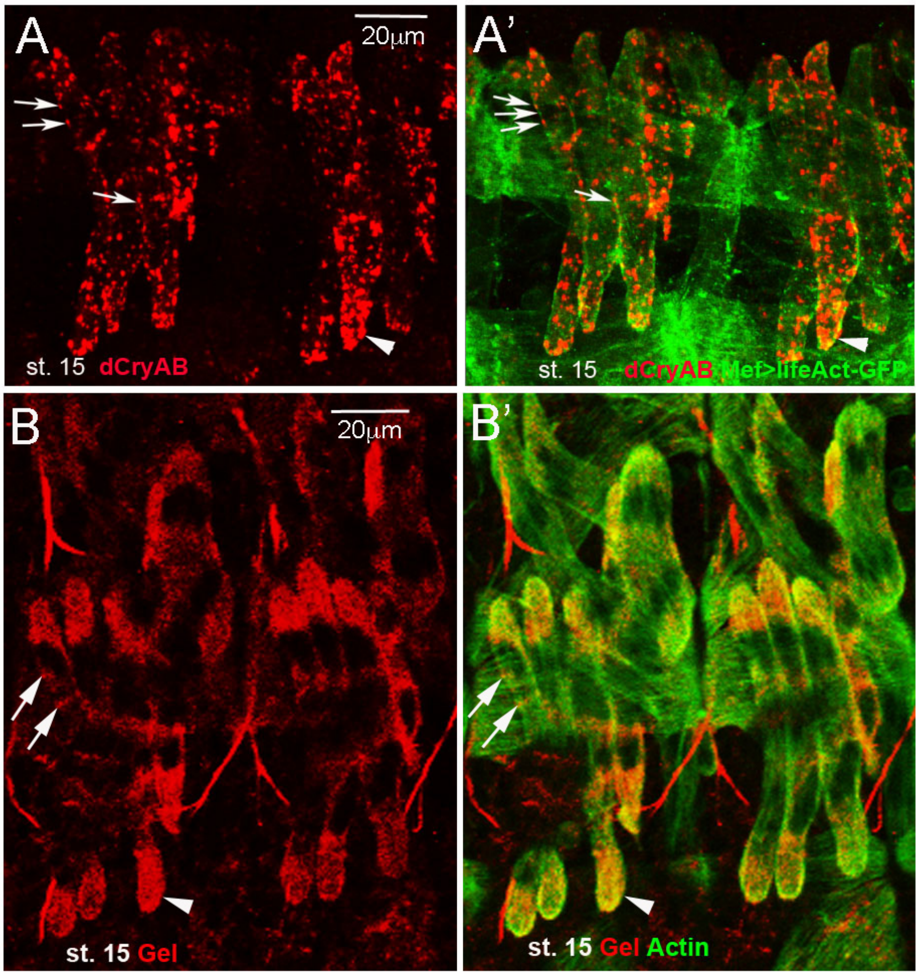
A
20μm
st. 15 dCryAB
A'
st. 15 dCryAB Mef>lifeAct-GFP
B
20μm
st. 15 Gel
B'
st. 15 Gel Actin

Figure S4

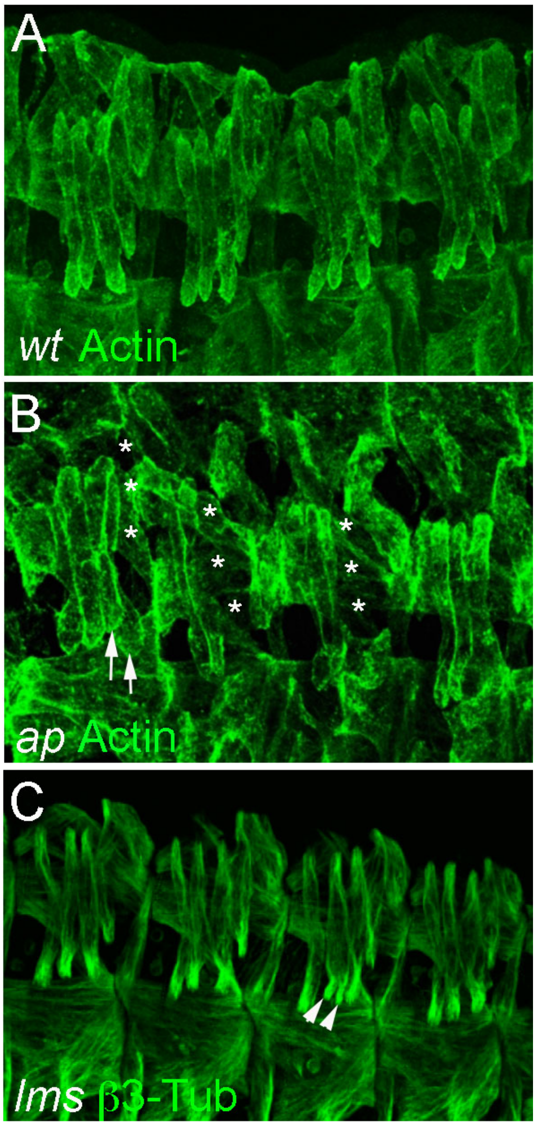

Supplement: Supplementary file 3 — Supplementary Information 1. [file 41598_2021_92506_MOESM3_ESM.pdf]
